# Introducing a Smart Toy in Eating Disorder Treatment: A Pilot Study

**DOI:** 10.3390/nu16040467

**Published:** 2024-02-06

**Authors:** Dimitri Chubinidze, Zhuo Li, Petr Slovak, Julian Baudinet, Emmanuelle Dufour, Kate Tchanturia

**Affiliations:** 1 Department of Psychological Medicine, Institute of Psychiatry, Psychology and Neuroscience (IoPPN), King’s College London, London SE5 8AF, UK; dimitri.chubinidze@kcl.ac.uk (D.C.); zhuo.li@kcl.ac.uk (Z.L.); julian.baudinet@kcl.ac.uk (J.B.); 2 National Eating Disorders Service, South London and Maudsley NHS Foundation Trust, London SE5 8AZ, UK; emmanuelle.dufour@slam.nhs.uk; 3 Department of Informatics, King’s College London, London WC2B 4BG, UK; petr.slovak@kcl.ac.uk; 4 Maudsley Centre for Child and Adolescent Eating Disorders (MCCAED), Maudsley Hospital, London SE5 8AZ, UK; 5 Department of Psychology, Ilia State University, Tbilisi 0162, Georgia

**Keywords:** eating disorders, anorexia nervosa, autism, emotion regulation, sensory sensitivity, technology-enabled interventions

## Abstract

Individuals with eating disorders (EDs) often encounter challenges related to body image, emotional, and sensory difficulties during nutritional rehabilitation. To address these challenges, a novel technology-enabled smart toy, Purrble, designed for immediate assistance in emotion regulation, is being explored. A mixed-method approach involving workshops, diaries, and focus groups was employed to examine the feasibility of Purrble as a therapeutic tool and its impact on participants’ daily routines, sensory experiences, and emotional states. The study results demonstrate the engagement and acceptability of this device. Qualitative analysis revealed that participants independently used and integrated Purrble into their emotional and sensory regulation practices. These pilot results support the potential for a shift in the delivery of adjunct therapeutic tools through technology, particularly for ED patients with complex presentations. Future research is necessary to further explore the psychological benefits of this intervention.

## 1. Introduction

Eating disorders (EDs) are complex conditions that impact an individual’s psycho-logical and physiological well-being. Often compounded by a range of comorbid condi-tions, EDs significantly influence both clinical practice and the quality of life for those affected [1,2]. A noteworthy comorbidity is that between EDs and the autism spectrum condition (henceforth ‘autism’, as preferred by the autistic community) [3], presenting unique challenges in daily routines for those undergoing treatment. This is particularly evident in the realm of sensory disturbances and difficulties in emotion regulation (ER) [4,5,6,7].

Research indicates a relationship between sensory processing and eating behaviors. Individuals with autism as well as with ED may exhibit heightened sensitivities in sensory domains such as smell, taste, vision, and texture [8,9,10,11,12,13,14,15]. This heightened sensitivity can be exacerbated in comorbid cases with ED and autism and can result in difficulties adapting to a treatment setting and the active avoidance of specific foods [16].

Emotion regulation capacity is also considered a crucial aspect of ED management. Challenges in recognizing, identifying and expressing emotions, which are exaggerated in the context of comorbid autism, can exacerbate the complexities of EDs [17,18,19,20,21,22]. Sensory disturbances, such as difficulties in interpreting and tolerating sensations, further influence emotion regulation. This is particularly relevant in EDs, where misinterpretation of bodily signals can lead to inappropriate emotional responses [23].

Consequently, for people with EDs, it may be beneficial to provide adjunct therapeutic interventions that specifically target ER, as well as sensory sensitivities. Accordingly, there is an expanding scope of research focused on exploring creative and innovative methods to augment talking therapies with evidence-based adjunct interventions [24]. In the novel clinical pathway for EDs and autism, known as PEACE (Pathway for Eating Disorders and Autism developed from Clinical Experience), these avenues are being explored (see [25] for details of this pathway). Within PEACE, adjustments have been made to the clinical environment, communication, and clinical practices to support patients’ sensory and emotional needs. This includes the development of psychoeducational materials and experiential activities aimed at enhancing sensory well-being [26,27]. Additionally, a sensory workshop has been developed to assist patients in creating a soothing sensory toolkit, which has been proven to be beneficial for patients [27,28]. It is worth mentioning that these adjunct interventions can benefit not only individuals with comorbid EDs or autism but every patient with an ED. The recognition of the need for additional activities and enhanced collaboration across clinical services has led to the acknowledgment that further development in these areas is essential. In response, our approach involved developing existing adjunct tools, such as the sensory well-being workshop, with the introduction of a technology-enabled tool—a socially assistive robot known as Purrble [29,30].

Purrble’s design is theoretically grounded in Gross’ extended process model of ER [31]. It specifically targets two distinct stages: (a) the attentional deployment stage, by redirecting the user’s focus away from the emotion-eliciting situation towards interaction with the device; and (b) the response modulation stage, by promoting downregulation through pleasant tactile interaction [32].

The soothing effects of interacting with Purrble are further heightened due to the device being modeled on human–animal interactions. Research in various domains suggests that tactile stimulation is an adaptive strategy for modulating stress responses. This mechanism is thought to be key in the emotion-regulating effects of human–animal interactions [33]. It is also hypothesized to play a role in ‘social touch’ [34] and in the calming benefits of animal-like robots [35]. Moreover, the effectiveness of human–animal interactions in promoting soothing and emotion regulation is further evidenced by animal-assisted therapies. These therapies have been recognized for addressing a variety of psychological needs, as indicated in several studies [36,37,38,39,40].

There is a growing body of research suggesting that Purrble can offer significant benefits by improving ER in young people [29,32,41] and in highly anxious university students [42]. Although there is emerging evidence suggesting that Purrble could support ER across the lifespan, its use in mental health clinical settings, particularly in the context of EDs, has not yet been explored.

The primary aim of this pilot study is to investigate (a) the level of engagement and acceptability of Purrble within intensive treatment programs for EDs and (b) the exploration of the perceived impact of this device on participants’ sensory and emotional well-being.

## 2. Materials and Methods

### 2.1. Participants

Participants in the study included adult and young patients with a confirmed DSM-5 [43] diagnosis of an ED (made by lead clinicians). All participants were recruited from ED services at the South London and Maudsley NHS Foundation Trust (SLaM) for young persons and adults. Anonymous feedback from participants was collected as part of routine clinical practice. The data generated for this study were approved by the SLaM Child and Adolescent Mental Health (CAMHS) Service Evaluation and Audit Committee (328) and by the SLaM Clinical Governance and Audit Committee (032019).

### 2.2. Intervention

**Figure 1:**
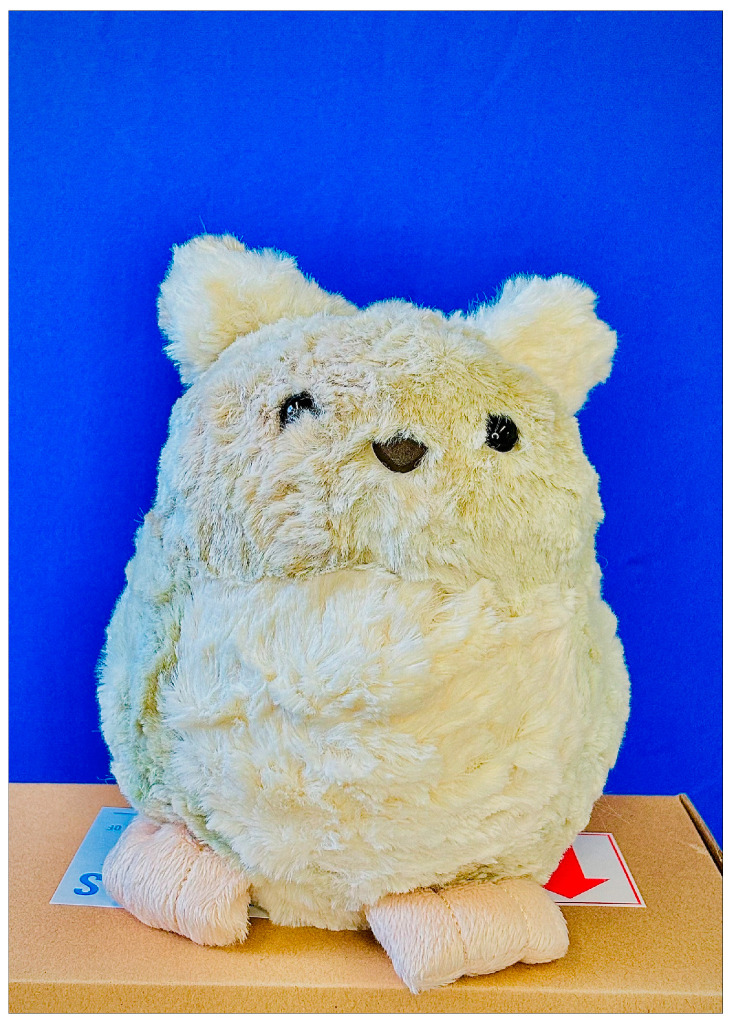
Intervention tool—Purrble.

Purrble is a compact, affordable sensory device shaped like a plush animal (Figure 1) designed to provide in-the-moment ER support in daily life [29,41]. The device is conceptualized as a creature that experiences anxiety, requiring care and comfort when agitated. Its emotional state is conveyed through a simulated heartbeat, created by inbuilt electronics that generate vibrations ranging from frantic and anxious to slow and calm. The toy’s heartbeat starts off fast and can be calmed through stroking, denoting a state of relaxation. While the calming process generally takes less than a minute, the duration can vary depending on the user’s interaction with the device.

Due to its sensory characteristics, including its soft and cuddly design, neutral colors, and gentle sensory feedback, such as soft vibrations and purring sounds, as well as its compact and portable size, Purrble showed promise as an addition to patients’ sensory toolkits. It can be used both in clinical settings and various other environments. Furthermore, this intervention is designed to require minimal clinical effort while offering additional therapeutic support and facilitating seamless integration across various clinical services.

### 2.3. Procedure

The study involved three stages: (1) a sensory wellbeing workshop (a single session) and Purrble distribution; (2) 10-day interaction period with the device; and (3) post-experience focus group.

Sensory well-being workshop and distribution of Purrble: Participants attended a one-hour session aimed to enhance their understanding of the sensory system and its role in self-regulation. It also provided strategies to improve sensory well-being and equipped participants with the tools and language to communicate their sensory needs. The workshop included a do-it-yourself (DIY) activity, where participants created a sensory item, such as a glitter jar or scented hand cream. The workshop was led by two or three facilitators and the first author. Details of the workshop’s procedure and protocol are available in the pilot evaluation [27,28]. At the conclusion of the workshop, the smart toy Purrble was introduced to the participants as a sensory tool. It was provided without explicit training in emotion regulation and distributed to each participant for personal use.

Ten-Day Interaction with Purrble Documented Through Diary Recording: Post-workshop, participants engaged with Purrble for a period of 10 days. They documented their daily interactions in a specially designed diary (see Supplementary Material Diary Template S1). This diary gauged the frequency of device use, its perceived benefits on emotional state improvement, and sensory sensitivity. Participants rated their experiences daily using a 10-point visual analogue scale. The diary also included a section detailing specific contexts or situations in which Purrble was used. Additionally, it featured an area for open-ended feedback, allowing participants to share reflections on their interactions with the device.

Focus Group Session: Following the 10-day experience, participants were invited to a focus group session to share their experiences. The discussion was also aimed at exploring any concerns, suggesting modifications, and discussing participants’ future intentions regarding the use of Purrble. The focus group, lasting 45 min, was facilitated by the first author and a member of the respective clinical team and audio recorded for analysis.

### 2.4. Analysis

Focus group recordings were transcribed verbatim by DC. Transcripts were read and re-read by authors DC and ZL to ensure thorough familiarization. In parallel, qualitative content from the participants’ 10-day diaries was compiled. An initial set of codes was generated by DC, based on the content of the transcripts, and it was evaluated by KT. The codes were then generated from the data using NVivo14 software. DC conducted qualitative content analysis [44] to identify potential themes, which were subsequently reviewed by ZL and KT. This review process involved evaluating how well the themes encapsulated the coded data and their reflection of the entire dataset. Themes are reported together with supporting quotes in the Section 3. Quotes from participants are anonymized using participant numbers (e.g., P1, P2 …). Quotes from focus groups are marked with ‘FG’.

In the Section 3, qualitative and quantitative findings are presented together, rather than separately, with quantitative data presented under relevant themes. This integrated approach will specifically highlight themes related to the usage patterns of the device and its perceived impact on ER and sensory sensitivity.

## 3. Results

In total, four workshops were conducted from June to September 2023, involving 26 patients across three ED services: inpatient and daycare programs for adults and the Intensive day Treatment Program (ITP) for young people (cf. [45] for programme details). The number of participants in each workshop ranged from 3 to 10. However, five participants (19.23%) did not return their diaries and were absent from the focus group. The reasons for non-participation among the five excluded participants varied. Three required admissions to a different ward due to worsening conditions, including self-harm and suicidal thoughts. One temporarily left London for vacation, and one chose to discontinue participation due to discomfort with Purrble, reminiscent of a negative experience with a similar toy. Clinical characteristics varied among these participants, with two having comorbid depression, one with comorbid anxiety, one with autism, and one participant having no comorbidities. Individuals who did not return diaries were excluded from the analysis, leaving a total of 21 cases.

Table 1 summarizes the participant demographics. The majority of the participants (*n* = 20) were female, with one individual identifying as non-binary. The mean age of participants was 21.9 years, with an age range of 13 to 37 years. The predominant ethnic background in the study was White British (*n* = 16), from other ethnic backgrounds (*n* = 5). Approximately 52.38% (*n* = 11) of the participants had a comorbid condition, and 38.1% (*n* = 8) had either a formal diagnosis of autism or high autistic traits, as assessed by the clinical team.

**Figure 2:**
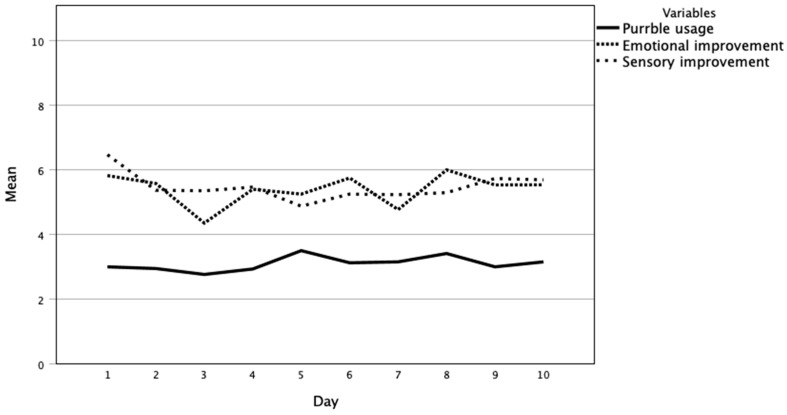
Trends in Purrble usage, emotional, and sensory improvements over 10-day experience.

On average, participants interacted with Purrble approximately 25 times over the 10-day period. While a rating scale was initially provided, the majority of participants chose to directly report the number of interactions with Purrble, and we estimated the daily usage based on the latter. The trend in their daily usage of Purrble and self-reported improvement in emotional state and sensory sensitivity over the 10-day period is reported in Figure 2. Four themes arose from participants’ qualitative feedback in diaries and focus groups, including the following: (1) engagement and relationship dynamics; (2) acceptability and ubiquity in daily activities; (3) anxiety, distress, and discomfort management; and (4) sensory tuning.

### 3.1. Engagement and Relationship Dynamics

This theme focuses on the dynamics of participant engagement with the intervention device. It explores the evolving nature of engagement with Purrble and the developing dynamics of the relationship between participants and the device. Additionally, this section investigates themes such as empathetic engagement, bonding, and perceived responsibility.

Figure 2 shows that overall, participants’ engagement with Purrble was consistent over the 10-day period. Qualitative analysis of diary entries and focus group discussions further identified that engagement evolved from curiosity into more focused and targeted interactions, particularly during moments requiring significant emotional support:

*I was probably using Purrble more at the beginning, but I think that was more exploratory at the start. And then, as the days went on, it shifted to more acute situations where I felt, ‘Okay, I really need to calm down’.*

[FG]

*Some days I used it more often than others, like when I encountered a stressful situation.*

[FG]

The interactions between participants and Purrble illustrate an evolving, empathetic relationship characterized by sentiments of responsibility, caregiving, and mutual emotional regulation. This dynamic extended beyond mere functional use, as participants perceived Purrble as an emotionally responsive companion. For example, participant 20 expressed a sense of caregiving responsibility:

*I sometimes feel like I have responsibility to look after it.*

[P20]

Further, participant 8 described a shared emotional state with Purrble, finding intrinsic calmness in soothing the toy:

*The only thing was that when Purrble’s heart rate increased, it made me feel quite anxious as if my toy was anxious. However, when it was calm, I fell into a rhythm of soothing it when it purred, and that was calming.*

[P8]

The depth of the bond between participants and Purrble is perhaps most poignantly illustrated in the creative expressions of the participants themselves. Participant 6 expressed their relationship with Purrble through a poem, reflecting the emotional connection and significance Purrble had during the experience. The poem reads as follows:

*I love my Purrble. His name is Arlo.*

*Now he comes everywhere I go.*

*I hold him close before a meal,*

*I love his softness I can feel.*

*First his sound and feel gave me joy,*

*Now just sitting next to me,*

*He is more than just a toy.*

[P6]

Despite participants gravitating towards this growing bond, it is important to acknowledge that not all perceived the empathetic relationship as beneficial. P18 voiced concerns about the potential emotional burden:

*It felt like you kind of had to look after another person, and sometimes you don’t feel like you can’t even look after yourself.*

[P18]

### 3.2. Acceptability and Ubiquity in Daily Activities

Through the analysis it emerged that Purrble was seamlessly incorporated into the participants’ everyday practices, both at home and on the ward/in clinic. Its presence in frequently used areas, such as living rooms and bedrooms, emerged as a consistent theme. For instance, a focus group participant mentioned, ‘*I generally used Purrble when I was in the living room, either watching TV or engaged in other activities. It was usually there on the couch, close by me*’. This sentiment was echoed by another participant who remarked, ‘*Purrble has been a constant presence in my living room*’ [FG], highlighting its pervasive role in their living spaces.

Additionally, participants highlighted Purrble’s role as a portable companion, providing support in various settings. This included scenarios such as ‘*walking around the house*’ [FG] and ‘*… using Purrble in the car while traveling*’ [FG].

The acceptability of the device is further illustrated by its incorporation into various routine activities such as leisure, study, work, or as a mealtime companion. Participants mentioned diverse reasons for using the device, including comfort, relaxation and improved concentration.

Purrble served as a companion during leisure activities, such as watching TV, and played a role in evening calming routines. Its effectiveness in offering companionship and comfort, especially during evening times, was emphasized:

*I normally use Purrble once or twice a day and mainly in the evening more often than throughout the day. So, especially before bed, it helped me calm down … Helping me in relaxing before going to sleep.*

[P16]

Focus group discussions further highlighted Purrble’s effectiveness as a sleep aid:

*I always find it difficult to sleep because my brain constantly races, going round in circles. Purrble gives me something to focus on, unlike when I focus on the TV, which often leads to me watching endless episodes of shows.*

[FG]

In addition to its role in relaxation, participants mentioned Purrble’s benefits in terms of heightening attentional focus and improving task management. A participant from the ITP noted its utility during academic activities:

*I was using Purrble when I was doing homework and stuff because I usually get distracted. So, by having Purrble, it kind of helped me counter that stressful situation because there was a lot to do. Having Purrble there was like having something to focus on.*

[FG]

In addition to predominantly home-based activities, other participants reported utilizing Purrble in clinical procedures and during psychological therapies, highlighting soothing support and facilitating verbalization during discussions of traumatic events:

*It [Purrble] had been helpful during therapy. Yeah, kind of like a sensory soothing toolkit while talking about stuff which is difficult.*

[FG]

*I get really distressed during NG [nasogastric feeding], so I use the Purrble. I mean, I quite like the fact that the heartbeat slowed down. It didn’t completely calm me down, but I think it did help. I think my heartbeat was really fast, and I think my heartbeat ended up slightly matching the heartbeat of the Purrble in a way.*

[FG]

### 3.3. Anxiety, Distress and Discomfort Management

This theme, together with those that follow, provides an examination of Purrble’s perceived impact in managing emotionally charged states and addressing sensory-related issues.

One of the most reported psychological states where participants utilized Purrble is anxiety. They emphasized its immediate use as a response mechanism to anxiety episodes:

*A lot of times, it’s still just hanging out when turned off, but then I’d actually seek it out for anxiety …*

[FG]

*Once the anxiety started, I would take Purrble. So, it was usually after the anxiety had begun.*

[FG]

*For me, it was mainly when I felt anxious, which happens often, especially in the evenings. During the initial days, I’d just pick Purrble up whenever I felt that way and soothe myself by stroking him.*

[FG]

One participant discussed the strategy of keeping Purrble close for immediate access in case of sudden anxiety:

*There were moments when I felt anxious, and I’d carry it around with me. The location varied based on whether I was alone in the house or not. If my housemates were home, I tended to only use Purrble in my bedroom. But when I was on my own, I’d have Purrble close by, just in case I started to feel anxious and needed immediate access.*

[FG]

While participants shared experiences of Purrble’s soothing effect in times of anxiety, some pointed out their need for more active forms of distraction during high-stress moments:

*Well, sometimes when I’m more stressed, I think I need more active distractions than just sitting with Purrble. But, you know, maybe sitting with Purrble would’ve been good.*

[FG]

*When I had a meeting, I was very angry and very distressed, and I just couldn’t. I didn’t want to soothe or comfort at all, so I was too angry, so I wasn’t able to reach out for Purrble.*

[FG]

Therefore, some participants, particularly in times of high stress, preferred more proactive coping strategies and found it challenging to calm down with Purrble.

Purrble demonstrated effectiveness in a variety of situations, including feelings of loneliness:

*When I woke up feeling anxious, Purrble helped me a bit; when I had an argument with my parents; when I felt sad or lonely; when I just needed comforting; needed something to distract me when I watched TV or read. When trying to get back to sleep.*

[P18]

Time spent in the ward can produce a sense of loneliness for inpatient service users. Participants reported the following:

*When I felt sad or sometimes even empty, I’d turn to him. I remember when I had a dog, cuddling with him always uplifted my spirits. So, with Purrble, it felt similar, like he’d cheer me up when I was down.*

[P7]

*I used Purrble when I was feeling sad, lonely, slightly anxious, when I wanted to feel comforted.*

[P3]

Participants also highlighted Purrble’s role in managing somatic conditions such as pain and shaking:

*After eating, I usually experience a lot of pain in my stomach. Purrble helped not only with my anxiety but also by providing a distraction. Just holding him kind of made me concentrate on him instead of the pain. My stomach gets really painful when I eat, so it helped to cuddle him against my stomach to take my mind off the pain.*

[FG]

*When I get anxiety, I get really bad shakes in my hands, and I found that just holding Purrble and feeling its vibrations helped to calm my shakes.*

[P1]

#### Mealtime Companion

One common experience was that Purrble helped participants with managing meal-related stress. An emotionally charged scenario commonly faced by individuals with ED involves stress and discomfort during mealtimes. Participants reported Purrble’s effectiveness in these specific contexts, including addressing pre- and post-mealtime challenges, as well as serving as a companion during meals.

A focus group participant shared their experience using Purrble to manage pre-mealtime anxiety:

*I’ve mainly used him before mealtimes … I’m getting used to reaching for him before a meal, especially as I approach my second mealtime. That’s when I tend to get most anxious, anticipating what’s to come.*

[FG]

Participants also reported significant challenges associated with post-mealtime discomfort and diverse emotional states, areas where Purrble provided relief:

*Yesterday was the first time that I actually went out to dinner and wasn’t sick afterwards. By the time we got home, my anxiety was getting worse and worse, to the point where I was close to having a panic attack. So as soon as I got home, I reached out for Purrble.*

[FG]

*Yes, I’ve used it after meals. In fact, that’s probably the most common time I’ve gone to it. That’s when I feel the highest level of stress.*

[FG]

*In my experience, it especially helped when I was overwhelmed, stressed, or had a lot of anxiety after meals. In these situations, it helped to ground me and bring my heart rate down, hearing and feeling my Purrble’s heart decrease and slow relaxed me and soothed me until I realized that my heart rate was slow, and I wasn’t really having thoughts, or I forgot what I was overwhelmed about.*

[P15]

In some instances, participants incorporated Purrble into their actual mealtime routine:

*I use it [Purrble] a lot before meals and sometimes during meals, so like have it next to me during the meal.*

[FG]

*It was helpful in the dining room to have something with you.*

[FG]

Participants also reported using the device during intense distress and feeling overwhelmed by sensory cues during the mealtime. A participant from the Daycare service provided a vivid scenario of using Purrble in a case of distress and sensory sensitivity:

*There was this one day where so much was happening. We were having a challenging meal …. Everything came to a head when a glass smashed. I was already cooking and feeling stressed, and then I knocked the glass over. I was so frustrated. I went upstairs, took a moment with Purrble, and then came back down. Only after that, I felt ready to clean up the glass and sort out the kitchen.*

[FG]

### 3.4. Sensory Tunning

The sensory characteristics of Purrble, such as its tactile and auditory feedback, played a significant role in ER. A participant from the focus group reflected on the calming effect of Purrble’s heartbeat:

*I found Purrble’s heartbeat incredibly calming. I knew beforehand that rhythmical things help ground me, so I expected the heartbeat to have a decent impact on me. But the softness of it was also comforting. I often cover my face when I’m anxious, and with Purrble, I found myself doing the same, holding him close to my face because of his soft texture. It reminded me of the comfort I get from blankets.*

[FG]

Emotions, with their intensity and character, bring forth varying sensory demands. A recurrent attribute emphasized by participants concerning Purrble was its lack of squishiness, particularly significant during intense emotional experiences. For example, Participant 7, during an emotionally challenging clinical procedure (i.e., Naso Gastric Feeding), expressed a need for tactile engagement:

*I realized I felt like I needed to squeeze something … But I felt like the problem was you couldn’t squeeze [Purrble], because I felt like if I squeezed it, it would have broken or something.*

To navigate this emotional distress, the participant intuitively combined Purrble with a dinosaur toy (‘teddy’) to fulfil this sensory need:

*What I would do is I would put the Purrble next to my teddy so I could squeeze something and then feel at the same time.*

[P7]

Sensory tuning, or the capacity to align with Purrble’s sensory output (i.e., heartbeat), emerged as a vital aspect for participants. The challenge of synchronizing Purrble’s heartbeat with their emotional states was both a therapeutic tool and a challenge. P2 accentuated the importance of personalization in managing the device’s heartbeat:

*I found it challenging to lower Purrble’s heartbeat. It would be ideal if there was an option to adjust settings, like deciding if or when the noises occur or controlling how long it takes for the heartbeat to slow down. I would prefer a shorter duration for the heartbeat to decelerate.*

The sense of achievement felt by participants for successfully calming Purrble is indicative of its therapeutic benefits. This engagement is characterized by a determination to regulate the toy’s heartbeat, as one participant expressed:

*Sometimes it made me quite determined to stop its heartbeat. When it started purring, then I’d also feel calm.*

[FG]

## 4. Discussion

The objective of this study was to investigate the engagement and acceptability of the socially assistive robot, Purrble, in people with EDs. A key novel aspect of this study was the introduction of the intervention in a mental health clinical setting for EDs. This is especially pertinent during the critical phase of nutritional rehabilitation, where the interplay of emotional regulation and sensory processing is vital in treatment efficacy and patient responsiveness. The incorporation of Purrble in such settings represents a progressive intersection of mental health technology and nutritional rehabilitation.

The pilot study’s findings suggest that participant engagement with the device was consistent throughout the 10-day experiential period. This level of engagement and acceptance aligns with prior deployments of the device across diverse groups, including children [29,32] and highly anxious university students [42].

Qualitative analysis further suggests that participant interactions with Purrble developed into empathetic relationships, characterized by feelings of responsibility and caregiving. This dynamic is indicative of mechanisms that potentially underpin sustained engagement with the intervention. For instance, portraying the device as an anxious entity in need of care seemed to foster a sense of relationship and responsibility towards the Purrble’s wellbeing, echoing findings from earlier studies involving Purrble [29,32,41]. However, it is crucial to acknowledge that while these relationships predominantly evoked positive feelings of comfort and care, they sometimes introduced an additional sense of responsibility. Such complex emotional responses hold particular importance in mental health clinical settings for patients with comorbid conditions, highlighting the necessity of a nuanced approach to the psychological effects of these interactions.

Additionally, our findings align with earlier research regarding the integration of the device in home environments, specifically within leisure and bedroom settings [46]. Uniquely in our study population, the Purrble device was seamlessly incorporated into the daily routines of participants, even in highly structured environments like ward settings and therapy sessions. This adaptability within both home and clinical contexts underscores the flexibility and potential of Purrble as a therapeutic tool in diverse settings and routines.

The patient-reported experiences in this study corroborate the immediate regulatory effects of the Purrble device evidenced in previous research [29,32,42]. However, our study’s participant group uniquely articulated a wide range of psychological states and scenarios in which they employed the intervention. These ranged from somatic symptoms such as pain and shaking to emotional and cognitive states, including anxiety, stress, panic, distress, concentration difficulties, overwhelming emotions, sadness, loneliness, anger, and sensory overload.

A particularly noteworthy application emerged in relation to mealtime routines. Participants in our study reported a significant positive impact of Purrble’s ability to provide sensory grounding and emotional regulation both before and after meals. The presence of Purrble was also noted to offer comfort and support during meals, a time often fraught with heightened anxiety for individuals with EDs. This finding underscores the potential of Purrble as a therapeutic tool not only in managing a broad spectrum of psychological states but also in specifically addressing the complex emotional and sensory challenges associated with mealtimes in this patient population.

Our findings support the effectiveness of the human–animal interaction design model in the context of ER, as hypothesized in a previous study [32]. They further confirm the calming effects of tactile engagement with animal-like robots [35], as well as the soothing and emotion-regulation benefits demonstrated by animal-assisted therapies [36,37,38,39,40].

Consistent with prior research [29,32], participants expressed appreciation for the sensory characteristics of the device, particularly the calming effects of tactile interaction. However, some participants highlighted difficulties in synchronizing their heartbeat with the device’s heartbeat, a challenge that became more pronounced during periods of high stress. This difficulty in achieving sensory tuning, or attunement, highlights a need for more customizable sensory features in therapeutic tools or varied sensory properties for more proactive engagement. These findings resonate with those from previous sensory well-being workshops [27,28], emphasizing the importance of creating personalized sensory toolkits. Such tools are essential to effectively address the diverse and specific needs of patients with EDs, enhancing the efficacy of sensory-based therapeutic interventions.

### 4.1. Implications

The feedback from participants in this pilot study suggests significant implications for broader research and clinical applications, particularly in the context of ED treatment programs. It could be particularly beneficial as a complimentary resource for individuals on waitlists for ED treatment programs. Given the high demand for specialized care in this field, patients often face prolonged waiting periods, sometimes exceeding 18 months, before they can access treatment [47]. This extended wait period highlights the critical need for innovative interventions bridging the care gap during these crucial waiting periods, offering immediate support and potentially enhancing long-term treatment outcomes.

### 4.2. Limitations

While the study offers valuable initial insights, more rigorous, large-scale research is necessary to ascertain the full extent and strength of these psychological effects. Key questions remain, particularly regarding the potential for these effects to facilitate long-term behavioral changes. Additionally, it is yet to be determined whether the reported effects are substantial enough to effectuate clinically significant changes in emotion-coping mechanisms and strategies. This highlights the need for further research to explore the longevity and clinical relevance of implications reported, thereby contributing to a more comprehensive understanding of the intervention’s impact in the context of mental health clinical settings and therapeutic practices.

Furthermore, in this pilot study, we have identified two methodological areas for improvement in future research. Firstly, our findings revealed a disparity between qualitative and quantitative data, particularly in terms of participant engagement with the device. To address this, future studies will employ more comprehensive measures to better capture the subtle changes in user experiences. The second aspect pertains to the diary design used for reporting interactions with Purrble. Participants opted to report their interactions directly rather than using the provided rating scale. This preference for direct reporting over the structured format indicates an opportunity to optimize the diary design for more user-friendly and accurate data collection in subsequent studies.

## 5. Conclusions

This study represents the first known investigation into the feasibility of a smart toy, Purrble, in mental health clinical settings. The exploratory data demonstrated how the intervention was integrated into the daily routines of participants in both home and clinical environments. The findings on engagement, acceptability, and qualitative effects are promising, demonstrating that participants were able to use the device and integrate it into their practices for regulating emotions and sensory sensitivity. Future research is needed to build upon these initial findings with larger studies that examine the psychological efficacy of this intervention. More broadly, pilot study results indicate the potential for a technology-enabled shift in the delivery of adjunct therapeutic tools, particularly for addressing the complex needs of patients with comorbid conditions.

## Figures and Tables

**Table 1:** Summary of Participants’ Health and Demographic Characteristics.

Variables	*n* = 21
Age, mean (SD)	21.9 (7.4)
Gender, female *n* (%)	20 (95.2%)
Ethnicity, *n* (%)	
White British	16 (76.2%)
White Other	1 (4.8%)
Black British	1 (4.8%)
Asian	1 (4.8%)
Mixed	2 (9.5%)
Diagnosis, *n* (%)	
AN restrictive subtype	17 (80.9%)
AN binge-purge subtype	3 (14.3%)
AN atypical	1 (4.8%)
Duration of ED in years, mean (SD)	4.8 (5.2)
Missing, *n* (%)	4 (19%)
BMI on admission, mean (SD)	15.85 (2.95)
Comorbidity, *n* (%)	
EUPD	2 (9.5%)
Autism (including trait and diagnosis)	8 (38.1%)
MDD	1 (4.8%)

Abbreviations: AN—Anorexia Nervosa; ED—Eating Disorder; BMI—Body Mass Index; EUPD—Emotionally Unstable Personality Disorder; MDD—Major Depressive Disorder; SD—Standard Deviation.

## Data Availability

The data presented in this study are available on request from the corresponding author. The data are not publicly available due to ethical considerations.

## Supplementary Materials

The following supporting information can be downloaded at: https://www.mdpi.com/article/10.3390/nu16040467/s1, Diary Template S1: 10-day diary template for documenting interactions with Purrble.
