# Supplementary material for: Introducing a Smart Toy in Eating Disorder Treatment: A Pilot Study
Source: Nutrients. 2024 Feb 6;16(4):467. doi: 10.3390/nu16040467 (PMC10891985; doi:10.3390/nu16040467)

DATE:
INITIALS:

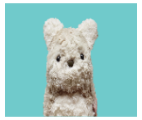

Welcome to Your Purrble Diary!

I'm Purrble, your cuddly friend, here to help when you feel emotional or sensory discomfort.

Like you, my heart races when I feel a lot. But when you hold and pet me, my heartbeat slows. You might find that yours does too!

In this diary, we'll track our 10-day journey together. Each day, think about how often, when, and why you used me. Also, consider how I affected your feelings and senses.

There's extra space for any other thoughts or feelings you want to share.

Ready? Let's begin!

In this **Daily Interaction Chart**, we're interested in how many times you use the toy each day and what emotional and sensory impact you experience. Please, for each day, provide the relevant number that best represents your answer.

	**1. How many times did you use Purrble today?**<br>1. Did not use<br>2. 1-2 times<br>3. 3-4 times<br>4. 5-6 times<br>5. 7-8 times<br>6. 9-10 times<br>7. More than 10 times	**2. How much did Purrble help improve your emotional state?**<br><br>Choose a number from 0 to 10, where 0 means no improvement and 10 means significant improvement.<br>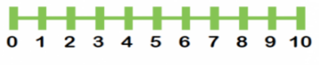 	**3. How much did Purrble help improve your sensory sensitivity?**<br><br>Choose a number from 0 to 10, where 0 means no improvement and 10 means significant improvement<br>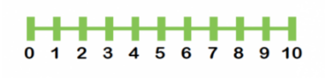
**DAY 1**			
**DAY 2**			
**DAY 3**			
**DAY 4**			
**DAY 5**			
**DAY 6**			
**DAY 7**			
**DAY 8**			
**DAY 9**			
**DAY 10**			

4. Throughout this period, please write down the situations in which you used Purrble or felt the need to. These situations might include feeling overwhelmed, having trouble focusing, experiencing anxiety, watching TV, during meal times, or having difficulty sleeping, among others. Feel free to add any other situations as well.

Extra Space for Feedback

This is your space to share any extra thoughts, feelings, or experiences from your time with Purrble.

Feel free to draw, write quotes, or express yourself in any other way you like. Suggestions for improvement are also welcome.

Our Journey Continues...

For your next visit, remember to bring this diary. We will hold a focused group discussion to share experiences and feedback. This is a great opportunity for you to share and listen to others' experiences with their Purrble companions.

Thank you for your time and contributions to this study!

Best,

Purrble

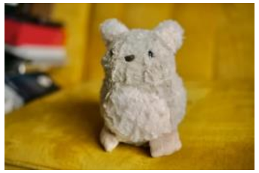

Supplement: Supplementary file 1 [file nutrients-16-00467-s001.zip › Diary Template S1. 10-day diary template for documenting interactions with Purrble.pdf]
